# A Computational Account of Borderline Personality Disorder: Impaired Predictive Learning about Self and Others Through Bodily Simulation

**DOI:** 10.3389/fpsyt.2014.00111

**Published:** 2014-08-27

**Authors:** Sarah K. Fineberg, Matthew Steinfeld, Judson A. Brewer, Philip R. Corlett

**Affiliations:** ^1^Department of Psychiatry, Yale University, New Haven, CT, USA; ^2^Center for Mindfulness, University of Massachussetts Medical School, Worcester, MA, USA

**Keywords:** associative learning, computational psychiatry, borderline personality disorder, embodied simulation, attachment

## Abstract

Social dysfunction is a prominent and disabling aspect of borderline personality disorder. We reconsider traditional explanations for this problem, especially early disruption in the way an infant feels physical care from its mother, in terms of recent developments in computational psychiatry. In particular, social learning may depend on reinforcement learning though embodied simulations. Such modeling involves calculations based on structures outside the brain such as face and hands, calculations on one’s own body that are used to make inferences about others. We discuss ways to test the role of embodied simulation in BPD and potential implications for treatment.

## Introduction

Social dysfunction in borderline personality disorder (BPD) is profound and persistent (1–3). In her memoir about BPD, Merri Lisa Johnson writes that having relationships was like “bleeding out,” evoking both the blurring of boundaries between herself and others and the bodily seriousness of the problem (4). Psychological formulations of BPD have also focused on the body.

Current theories describing BPD are complex and treatment paradigms are time intensive and expensive. Given the large psychological and healthcare burden of BPD, theoretical and treatment innovations are essential (5–7).

We aim here to present a hypothesis: that people with BPD have deficits in embodied simulation, which is a way of computing information about others that uses data from ones’ own body (see further explanation below). We discuss the evidence for this position, possible experimental approaches to testing it, and potential implications for treatment.

## Computational Psychiatry

We outline our hypothesis using a computational psychiatry approach. Given that this is a new but growing field, it is worth outlining what we mean by computational psychiatry. Computational psychiatry involves applying advances in computational neuroscience to psychiatry (8–11). More specifically, we think of computational psychiatry as an extension of cognitive neuropsychiatry (12). Cognitive neuropsychiatry involves specifying a cognitive system or process with relevance to a psychiatric symptom and examining how aberrations of that system or process might give rise to the symptom. For example, monitoring of inner speech may be relevant to auditory verbal hallucinations (13). Computational psychiatry develops this approach more formally, specifying mathematically how a problem is solved and then describing and quantifying how mental symptoms may arise in departures from that formalization. Those departures are delineated in terms of model parameters and the magnitude of change in those parameters required to engender mental symptoms.

Computational psychiatry may be useful in generating and testing hypotheses about mental illness. Furthermore, it may be possible to use computational psychiatry to generate and apply more data-driven nosologies for diagnosis and treatment. For example, by specifying which processes (neural, psychological, and behavioral) might be driving an individual’s problems, we can use computational psychiatry to allocate them to particular treatment strategies. More deeply, it may be possible to use computational neuroscience to bridge mental symptoms with neural processes. We make this assertion by considering David Marr’s levels of analysis (14), which we think may be key to achieving what Fodor thought to be impossible (15), a consilient understanding of cognition and its implementation in the brain.

After David Marr and Thomas Poggio, we believe that the computational problems solved by a system (e.g., a brain) (14) and their malfunctions (e.g., in mental illnesses like BPD) can be described at three levels of analysis:

(1) Computational – what are the representations being used by the system to solve the problem?
(2) Algorithmic – how are those representations combined and manipulated to solve the problem?
(3) Implementational – how does the hardware (neurons, circuits, brain, and body) implement the computations to achieve the end?

Thus far, most computational psychiatry has been algorithmic. It has used that level of analysis to discover what is happening at the implementational (neural) level and how that implementation may be disrupted in illness. We find this to be a worthy and efficient approach. But it is not the only approach. It is also, we feel, not the only way that a computational framework might be useful to psychiatry.

Here, we begin at the computational level (14), roughing out the problem at hand like a sculptor (16) – seeing whether there is any use for a computational explanation of BPD at the representational level. We think the utility of this approach is at least twofold: first, we can generate a language to explain to patients what their problems are and how they might be dealt with and second, we can generate testable hypotheses that will increase our understanding of BPD and hence facilitate better treatment.

As we progress in our work, we aim to fill in the finer and finer detail.

We believe the computational problem with BPD involves predicting the intentions of others using our own experiences as a template. We do not present any formal simulations nor any analysis of behavioral data in these computational terms – this will be the topic of our future work. We aim for an algorithmic, equation driven, explanation for the symptoms of BPD. This may be useful clinically, since parameter values from the computational model may be useful in clustering groups of patients into pathophysiological categories (10, 17, 18). Such clustering may be a useful therapeutic guide toward treatments that addressed underlying pathophysiology.

In this hypothesis and theory article, we will state our predictions. Our subsequent work will test those predictions.

## BPD and the Body

Many have ascribed BPD pathology to disturbance in early attachment relationships (19, 20), specifically disruptions between mother and infant occurring at the skin surface, which regulate bodily needs such as being nourished and soothed (19). Current diagnostic criteria for BPD also emphasize the physical self: feelings of (physical) emptiness, dissociation (feeling physically apart from the current situation), self-harm, and suicidality. Studies of tactile experience in BPD have found decreased pain sensitivity (21–24), and change in discussion of scars (25) and tattoos (26). Psychotherapies for BPD already engage the body: DBT begins with physical safety assurances and continues with body-grounded mindfulness practices (27), and schema-focused therapy asks patients to trade chairs to physically take another’s perspective (28).

## Embodied Simulation Theory

**Figure 1:**
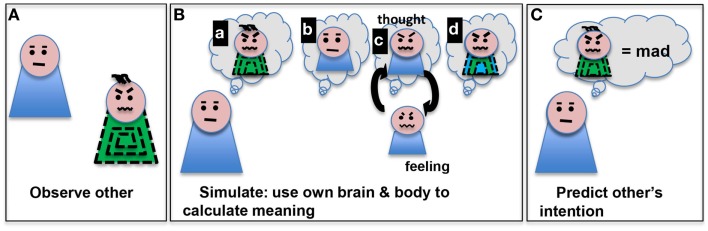
**Embodied simulation is a computational path from observing an action (such as scrunched eyebrows and mouth) in someone (A) to making a prediction about that action’s meaning (mad) (C)**. We depict several aspects of this process (as sequential steps here for clarity) **(B)**: (a) imagining the other’s action, (b) imagining oneself, (c) thinking of one’s own experiences with that action and low level activation of motor program to do that action, and (d) development of a model based on the observed other and personal simulated thoughts/feelings. The model can be used to predict the other’s intentions **(C)**.

In a ground-breaking 1996 paper, Vittorio Gallese and colleagues described the observation that certain neurons in the macaque area F5 (ventral pre-motor cortex) are activated both by doing an action and by observing someone else do the same action (29). They have termed these cells “mirror neurons.” Though single cells with mirror properties have not been much studied in humans (30), a system of brain regions with mirror properties have been mapped (31). These include pre-motor cortex, supplementary motor areas, primary somatosensory cortex, inferior parietal cortex, anterior cingulate cortex, and insula (31). Experiments transiently blocking mirror-neuron regions in human with transcranial magnetic stimulation (TMS) have shown deficits in mirroring activity (32, 33). These observations led to an embodied simulation explanation of the roles these mirror-neurons might play in cognition (34). This explanation suggests two important things (see Figure 1); first, observing others performing actions engages our own representation of those actions (mirroring) and second, we make inferences of others intentions using that evoked representation of our own action. The embodied simulation hypothesis takes an “extended mind” position (35): that the mind does computations in the brain, body, and perhaps the near environment. To make these inferences, we simulate what our own intention would have been, given their action kinematics and the local context (36). Hence, we use our representation of bodily self to make predictions about others, based on our own beliefs about intention, agency, and responsibility.

Of course, mirroring in neural activity may be a product of simpler associative learning mechanisms (37). We focus here on the possibility that these learning mechanisms may have gone awry in BPD and how that might manifest in terms of disrupted inference about ones’ body and other people.

Learning environmental contingencies (cues that predict rewards) and social contingencies (whether to trust someone’s advice) can be described by the same mathematical models (38). Causal model theories are an important new direction in cognitive science, based in Bayesianism. Thomas Bayes’ doctrine of probabilities formalizes reasoning about data using hypotheses and captures the probabilistic nature of many of the tasks faced by organisms. Organisms must predict their environments and respond appropriately. Thus, the brain forms and maintains a set of prior expectancies (predictive associations) about incoming stimuli in order to minimize uncertainty about subsequent inputs. This prediction error minimization is evident in hierarchical neuroanatomy down to the level of single neurons (39, 40).

The development of associationism has highlighted the value of a reductionist approach to the mind and brain to explain complex cognitive processes. As we predict our world and coordinate adaptive responses, prediction errors generated in response to a maladaptive environment can lead to strongly learned and difficult to change associations that persist into new circumstances. Also, people who erroneously signal prediction errors can learn strong (sometimes maladaptive) associations that may manifest as symptoms.

These predictive models are likely developed at least in part by embodied simulations (41, 42): we make inferences regarding other people’s intentions by generating a model of our own intentions in similar contexts and using that model to predict what other people will do (36). Object relations theorists describe these predictions about new people in terms of referencing, or perhaps even overlaying, known models of people (“objects” developed through early experience) onto novel people (20). Associative learning theory agrees that social predictions would be based upon past experiences. If those predictions fail, healthy adults can update their beliefs into a more accurate model. It may be that the social difficulties that attend psychiatric illness represent difficulty with this process of making, testing, and updating accurate social models.

Although others have raised concern about whether embodied simulation is used just for mimicry, for some form of understanding others, or for mentalizing in the sense of being able to describe another’s state of mind [for reviews of this issue, please see Ref. (35, 43)], leaving this debate unresolved for the time being, we can, however, assume that any of these uses of embodied simulation are important for social cognition, and that therefore deficits in embodied simulation do impact on real world social functioning.

## Computational Models of Learning: Relevance to BPD

Computational models of learning distinguish between two neuro-computational systems that can control behavior (44). First, there is a model-free system that learns and caches the values of being in particular states. In this system, behavior is guided toward the choices that lead to subsequent states with the highest cached value. This system is robust because it is computationally simple, but it is insensitive to sudden changes in those values (e.g., if a food source is poisoned or a relationship becomes toxic). This cached value system has been associated with the function of the dorsolateral striatum via human imaging studies, recording and lesion studies in rodents (44). On the other hand, there is a computationally intensive Bayesian tree-search mechanism that constructs model of the environmental contingencies and explores those models to arrive at the best course of action. This system has been related to the functioning of the pre-frontal cortex (44). Behavioral control is ceded to the system that is most certain about the next option to take. In a stable predictable world, this is most often the computationally efficient striatal habit system.

Tony Dickinson and Bernard Balleine have proposed an associative-cybernetic model of learning that foreshadowed this computational framework. The associations between cues, rewards, and actions we highlighted above may well be the representational basis for model-based and model-free learning (45, 46).

Aberrant association as a result of inappropriate social interaction and feedback will bias the competition between these systems and the simple, robust habit system will be favored. We argue that when model-based mentalizing fails people with BPD learn inappropriate (but computationally efficient) social habits. For example, someone with BPD might have learned that other people are unpredictable and untrustworthy, and they would find it difficult to update this belief, even in the face of confounding experience.

Fonagy has argued that in BPD, genetic and early environmental factors undermine mentalized affectivity – the ability to represent either people’s emotional states (a second order belief task). The stress and discomfort associated with this disruption (according to Fonagy) disrupts the development of cognitive control (47). It also contributes to poor attachment and an interactive cycle of further weakening attachment (since poor affect-recognition can make sensitive caregiving more challenging). Disorganized attachment also disrupts self-concept, creating incoherence, and splitting that is hard to manage and leads to frantic attempts to avoid abandonment, as well as the characteristic intense pattern of interpersonal relationships that escalate rapidly (47).

Fonagy points to the importance of the opioid and dopamine systems in developing attachment (47). He highlights the work of Jaak Panksepp in identifying a common neurobiology for mother–infant, infant–mother, and romantic attachments that involve opioids expressed in the frontostriatal dopaminergic circuitry (48). This same circuitry is involved in belief-based inference – in particular, that circuitry is engaged when evidence violates expectation (49) and in first-time mothers when they see an image of their own infant. People with disorganized attachment have a sensitized stress response (47) and are less likely to engage this inference circuitry and mentalizing processes when viewing pictures of their own child in distress (50, 51).

We posit that the balance between model-based and model-free reinforcement learning, mediated by uncertainty-based competition (44) is instructive here. Due to deficits in dopamine and opioid signaling (resultant from genetic and environmental insults) BPD individuals have attachment issues. Because of these issues, they find it hard to learn a self-concept and to use that self-concept to mentalize (using the more computationally intensive and uncertainty sensitive model-based learning system in pre-frontal cortex). This generates stress that biases processing even more in favor of the habitual model-free controller. In the parlance of Regina Pally, through the model-free system, the individual learns a pattern of predictive responses in infancy that are recapitulated later in life (52, 53). The enhanced role of striatally mediated, relatively inflexible model-free responding is also consistent with the increased susceptibility to substance abuse in this population – since addictive substances also hijack this brain and behavioral circuitry.

Habitual model-free responding is enhanced with decreased opioid signaling in PFC (54) or with increased striatal dopamine (55) signaling in experimental animals. We believe these two processes are candidate pathophysiological markers in BPD and they highlight potential therapeutic avenues for future work.

Bringing these ideas back to our original predictive coding scheme – the control of behavior can be model-based (flexible and representationally rich) or model free (inflexible and representationally lean). Which system controls behavior depends on the systems’ relative degrees of certainty about upcoming decisions (56). Within the predictive coding scheme that certainty is conveyed as precision (57, 58), the degree of confidence, we have in prediction errors (mismatches between expectation and experience). Dopamine enhances precision in this scheme, much like the uncertainty that biases instrumental control (trade-off between the two control systems – model based vs. model free).

## Hypothesis: BPD Social Dysfunction Relates to Difficulty with Embodied Simulation

In addition to feeling personally disembodied, as described in early psychological theory and the DSM, people with BPD may also have difficulty imagining embodied others – using the flexible, model-based pre-frontal tree-search system.

We believe that BPD pathology may arise from a deficit in representing bodily self. It may also involve a dysfunction in associating our action representations with those of others that we observe (59). People with BPD may have difficulty inverting representations of personal actions to make correct inferences about others (36). These predictions can be tested in a computational psychiatry framework that combines theoretical predictions with neural and behavioral data. It may be that each of these dysfunctions represents an endophenotype or sub-population of patients who meet BPD diagnostic criteria. Knowing more about the mechanisms that drive their particular dysfunction will allow us to devise targeted therapeutic approaches and allocate individuals toward those approaches based on their particular difficulties.

We predict that the diminished subjective sense of embodied self in BPD is associated with decreased use of embodied predictive simulations to generate beliefs about others, and decreased use of prediction errors to update those beliefs. Without the ability to incorporate new information about other people, social models may be applied inflexibly, leading to confusing and unstable interpersonal interactions.

## Experimental Methods: Linking Psychological Theory to Social Learning and Embodied Simulation in BPD

Combining neuroscience theory (including mirror-neuron theory) and psychoanalytic theory is not new. To pick just a few among many examples, Nicola Diamond considers Winnicott’s mirroring between mother and baby against the phantom body of Ramachandran (60), Vittorio Gallese considers mirror neurons as a potential substrate for developing interpersonal attunement (61), and Shantel Ehrenberg relates the process of learning dance movements in the studio to mirroring oneself and others in the frameworks of Lacan and the mirror-neuron system (62).

Nonetheless, these ideas are not widespread on the ground among BPD clinicians and researchers. The ability to enact social simulations has not been directly assayed in BPD patients, but several related functions have been studied. Zhao *et al*. have reviewed recent work describing self in mental illness, including BPD (63). Women with BPD had difficulty in giving detailed accounts of their own lives, and this correlated with difficulty figuring out social problems (64). Another study found that adolescents with BPD completing a social task “hypermentalize”; they indulge in too many hypotheses about others’ intentions with counter-productive results. They are ultimately unable to make *useful and use-able* hypotheses (65). BPD subjects also had more trouble than did control subjects with cooperating in an economic game where sharing might be advantageous (66). Tasks that engage social processes from recognizing facial emotions (67) to looking at (versus intentionally distancing from) negative social images (68) to activating empathy (69) all resulted in *decreased* activity of mirror-neuron regions in BPD versus control subjects. We might even speculate that dissociation could represent failure to be able to simulate very upsetting situations *within oneself*, perhaps giving rise to the subjective sense of *not being in one’s own body*.

We predict that a more globally effective intervention would engage the brain regions that integrate new data into preexisting models. For example, dopamine levels in pre-frontal cortex can increase model-based reinforcement learning in rodents (70). Also, Tim Behrens, Laurence Hunt, and Matthew Rushworth (38, 71) have found that developing a model about how much to trust another person’s advice engages the superior temporal sulcus and temporoparietal junction (mirror-neuron regions), and that weighing the advice with one’s personal factual models engages the ACC and ventromedial pre-frontal cortex.

Novel therapies for BPD may aim to directly alter the activity of these brain regions. For example, a recent study found that people’s calculations about sharing money with a partner could be titrated by direct current stimulation of the right lateral pre-frontal cortex (72). These non-invasive safe techniques, practiced to effect, may have promise for modulating both mirror neuron and motor regions.

Furthermore, psychological tasks may allow us to quantify clinical progress. For example, the social valuation task developed by Behrens, Hunt, and Rushworth described above allows use to quantify relative use of social data (38, 71). Furthermore, holding a specific facial emotion modulates our reaction time to understand novel emotional sentences (73). These and other measures could be used to assess the efficacy of novel socially directed interventions.

Finally, embodied robots, programed with the algorithms we outline, may be used to explore our hypotheses. There are already studies of robots interacting with one another (74). Aberrant prediction errors (and hence inappropriate association) can be introduced into a single robot. This engenders inappropriate behaviors by disrupting the learning and application of top-down priors (75). The same processes could be examined in two interacting robots. It would be useful to study the effect of different initial conditions and biases in the interactant (second robot) as well as the effect on the interactant of engaging with the BPD model robot.

Further research could help to clarify the relative contribution of specific cognitive mechanisms to symptom profiles for individual patients, and to develop (or assign already available) focused treatments to our patients.

## Social Learning in Psychiatry

Of course, BPD is not the only mental illness characterized by social difficulties. Autism spectrum disorder (ASD) and psychotic illnesses are other salient examples of illnesses in which social interactions are profoundly challenging. In the spirit of the Research Domain Criteria initiative, it is important for us to consider whether the model that we have outlined is specific to BPD or may be applied to social dysfunction more broadly. Are we addressing something that occurs across diagnoses and might even speak to the debilitating and stigmatizing effects of having a diagnosis of mental illness?

Inappropriate habit learning has been implicated in psychotic illness and likewise, excessive precision (or certainty) has been associated with ASD (76). Both of these illnesses then would involve social dysfunction because of impaired model-based pre-frontal learning in the context of spared and perhaps even enhanced striatal responding. However, we feel that the manner in which the dysfunction arises is critical. While trauma may play a role in the genesis of psychosis, we feel there is something specific about the perturbed infant-parent interaction that culminates in BPD. By gathering the same data and performing the same model analyses in groups from each diagnostic category, we will be able to assess whether this prediction holds.

## Concluding Remarks

Our prediction is that people need to be able to make and use predictive models to do well in social interactions. Therapies that engage one’s own body (such as the mindfulness modules of DBT) may help to repair embodied simulations. This could be a useful pre-requisite to treatments that directly promote flexibility and precision in social simulations. Predictive models of social situations depend on understanding others’ emotions and actions by simulating them in one’s own mind *as though they are actually happening* in one’s own body. This may only partially overlap with being able to verbalize other’s intentions. Feeling those intentions is also key. Re-acquainting oneself with own body experience will facilitate simulating the experiences of others. Subsequently, when exposed to other patients in group therapy, our “body-centered patients” may be better able to use physical and verbal emotional displays from others to simulate and update their social predictions.

## Conflict of Interest Statement

The authors declare that the research was conducted in the absence of any commercial or financial relationships that could be construed as a potential conflict of interest.
